# Generation of macrophage containing alveolar organoids derived from human pluripotent stem cells for pulmonary fibrosis modeling and drug efficacy testing

**DOI:** 10.1186/s13578-021-00721-2

**Published:** 2021-12-18

**Authors:** Hye-Ryeon Heo, Seok-Ho Hong

**Affiliations:** 1 Department of Internal Medicine, School of Medicine, Kangwon National University, Kangwondaehakgil 1, Chuncheon, Gangwon-do 24431 Republic of Korea; 2 Institute of Medical Science, School of Medicine, Kangwon National University, Chuncheon, Republic of Korea; 3 Environmental Health Center, Kangwon National University Hospital, Chuncheon, Republic of Korea

**Keywords:** Alveolar organoids, Macrophages, Human pluripotent stem cells, Pulmonary fibrosis

## Abstract

Macrophages are a central immune component in various types of in vitro human organoid systems to recapitulate normal and pathological development. However, to date, generation of human alveolar organoids (AOs) containing macrophages for use as a pulmonary fibrosis (PF) model and drug efficacy evaluation has not been reported. Here, we generated multicellular alveolar organoids (Mac-AOs) containing functional macrophages derived from human pluripotent stem cells based on stepwise direct differentiation by mimicking developmental cues in a temporally controlled manner. Derived Mac-AOs contained the expected range of cell types, including alveolar progenitors, mesenchymal cells, alveolar epithelial cells (type 1 and 2), and macrophages. Treatment with transforming growth factor (TGF-β1) induced inflammation and fibrotic changes in Mac-AOs, offering a PF model for validating the therapeutic potential of new drugs. TGF-β1-induced fibrotic responses and collagen accumulation in these Mac-AOs were effectively ameliorated by treatment with Pirfenidone, Nintedanib, and NP-011 via suppression of extracellular signal-regulated kinase signaling. To the best of our knowledge, this is the first report to provide non-epithelial functional macrophage-containing human AO system, which will better recapitulate the complexity of in vivo alveolar tissues and advance our understanding of the pathogenesis and development of effective therapies for PF.

**Supplementary Information:**

The online version contains supplementary material available at 10.1186/s13578-021-00721-2.

Dear editor,

Pulmonary fibrosis (PF) is a chronic and irreversible respiratory disease with poor prognosis and no effective treatment, highlighting the need to identify novel and effective therapeutic drugs for the treatment of PF [1]. Recently, the development of in vitro three-dimensional alveolar organoid (AO) system using human pluripotent stem cells (hPSCs) has attracted great attention compared to conventional monolayer cultures for studying early lung development, modeling disease, screening for novel drugs and the reciprocal interaction between type 2 alveolar epithelial cells and the niche in a pathological development of PF [2, 3]. One shortcoming of these organoids, however, is the lack of crucial immune cell components, such as macrophages and neutrophils, that play a pivotal role in the pathological development of PF and other respiratory diseases. This deficiency limits our capacity to model PF and subsequent drug screening approaches using these AOs.

In the present study, we describe a protocol for the generation of multicellular AOs, which contain both functional AECs and macrophages derived from hPSCs. The method is based on our optimized stepwise direct hPSC differentiation via mimicking of developmental cues in a temporally controlled manner [4, 5]. These macrophage-containing AOs (Mac-AOs) exhibit phenotypic and genetic resemblance to in vivo human alveolar tissues and recapitulate critical PF pathological features, including inflammation, fibrosis, and collagen accumulation, following transforming growth factor (TGF)-β1 treatment. We further evaluated these fibrotic Mac-AOs for their use in evaluation of the therapeutic efficacy of drugs including PFD, Nib, and NP-011 (modified milk fat globule-EGF factor 8 protein) for the treatment of PF.

## Generation of functional macrophages from hPSCs

**Fig. 1:**
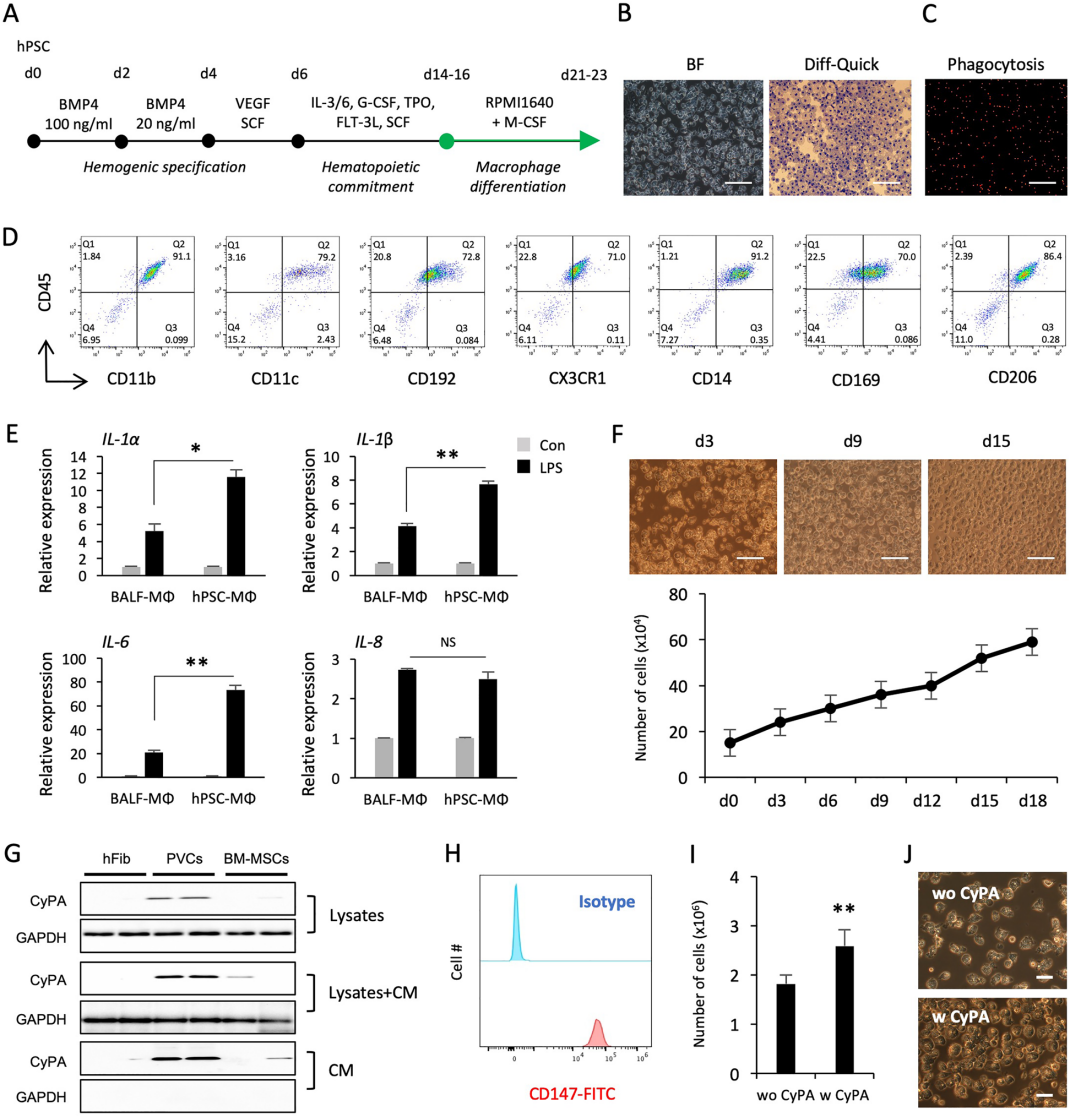
Generation and functional assay for macrophages from hPSCs. **A** Schematic overview of the stepwise direct hematopoietic and macrophage differentiation protocol from hPSCs. **B** Representative BF and Diff-Quick staining images of macrophages. Scale bars, 100 μm. **C** Phagocytosis of red fluorescein-labeled latex beads by hPSC-Macs. Scale bar, 100 μm. **D** Flow cytometry analysis of the expression of typical macrophage markers on hPSC-Macs. **E** The expression levels of inflammatory cytokines in macrophages after LPS stimulation for 24 h. Human macrophages obtained from BALF were used as a positive control. **F** Numbers of accumulated macrophages were counted every 3 days for up to 18 days, and the representative light microscopy images from different time points are shown. Scale bars, 100 μm. **G** CM and lysates were harvested from human PVCs and BM-MSC cultures and analyzed for expression of CyPA by western blot. Human skin fibroblasts (hFib) were used as a negative control. **H** Expression of CD147 in hPSC-derived hematopoietic progenitors was analyzed by flow cytometry. **I** Effects of CyPA on differentiation of macrophages. Hematopoietic progenitors were seeded at a density of 6 × 10^5^ cells/well of 12 well plate and cultured in the presence and absence of CyPA (50 ng/mL) for 8 days. **J** Representative images of macrophages cultured in the presence and absence of CyPA. Scale bars, 20 μm. Data are presented as mean ± s.d. *p < 0.05, **p < 0.01

Given the implications of macrophages in regulation and development of various respiratory diseases, we hypothesized that Mac-AOs are more relevant to model PF and evaluate the anti-fibrotic effects of PFD, Nib, and NP-011. Based on our robust serum-, feeder-, and xeno-free hematopoietic differentiation method, we harvested hematopoietic progenitor cells between days 14 and 16 of differentiation and cultured them for 7 days in macrophage induction medium (Fig. 1A). Human PSC-derived macrophages (hPSC-Macs) exhibited a morphological appearance similar to that of primary human macrophages and were capable of effective phagocytosis (Fig. 1B and C). Flow cytometry analysis showed that hPSC-Macs express high levels of macrophage-specific markers CD11b/c, CD192, CD14, and CX3CR1, indicating that these cells are phenotypically similar to mature human macrophages (Fig. 1D). Interestingly, human alveolar macrophage-specific markers, such as CD169 and CD206, were also highly detected on their cell surface (Fig. 1D). The inflammatory responses of hPSC-Macs were compared with primary human lung macrophages obtained from human bronchoalveolar lavage fluid (BALF-Macs). The expression levels of pro-inflammatory cytokines (*IL-1α/β*, *IL-6*, and *IL-8*) were upregulated by stimulation with lipopolysaccharide (LPS) in both hPSC- and BALF-Macs and were generally higher in hPSC-Macs compared to BALF-Macs (Fig. 1E). Similar results were obtained using macrophages derived from three additional hPSC lines (CMC003, CMC009, and CMC011; Additional file 1: Fig. S1). Long-term cultivation of hPSC-Macs in the presence of macrophage colony-stimulating factor resulted in continuous expansion of these cells for more than 2 weeks (Fig. 1F).

We have previously shown that perivascular cells (PVCs) enhanced the production of hematopoietic progenitor cells and macrophages from hPSCs via paracrine actions [6]. Using liquid chromatography tandem mass spectrometry, we have identified cyclophilin A (CyPA) in PVC-conditioned medium (CM) as a putative factor for promoting hematopoietic cell output from hPSCs [7]. Thus, we sought to determine whether CyPA exerts a positive influence on the macrophage yield from hPSCs. The protein level of CyPA was higher in CM and lysates from PVCs than in those from bone marrow-derived mesenchymal stem cells (BM-MSCs) (Fig. 1G). CD147, a signaling receptor for CyPA, was also highly expressed on the surface of hematopoietic progenitor cells (Fig. 1H). Notably, treatment with CyPA (50 ng/mL) during macrophage differentiation significantly increased the production of macrophages without alteration of frequencies compared with untreated control cells (Fig. 1I and J; Additional file 1: Fig. S2). These results indicate that hPSC-Macs phenotypically and functionally resemble primary human macrophages and may contribute to the development of a more relevant in vitro human AO model for PF and drug screening.

## Effects of PFD, and Nib, and NP-011 on fibrosis in Mac-AOs

**Fig. 2:**
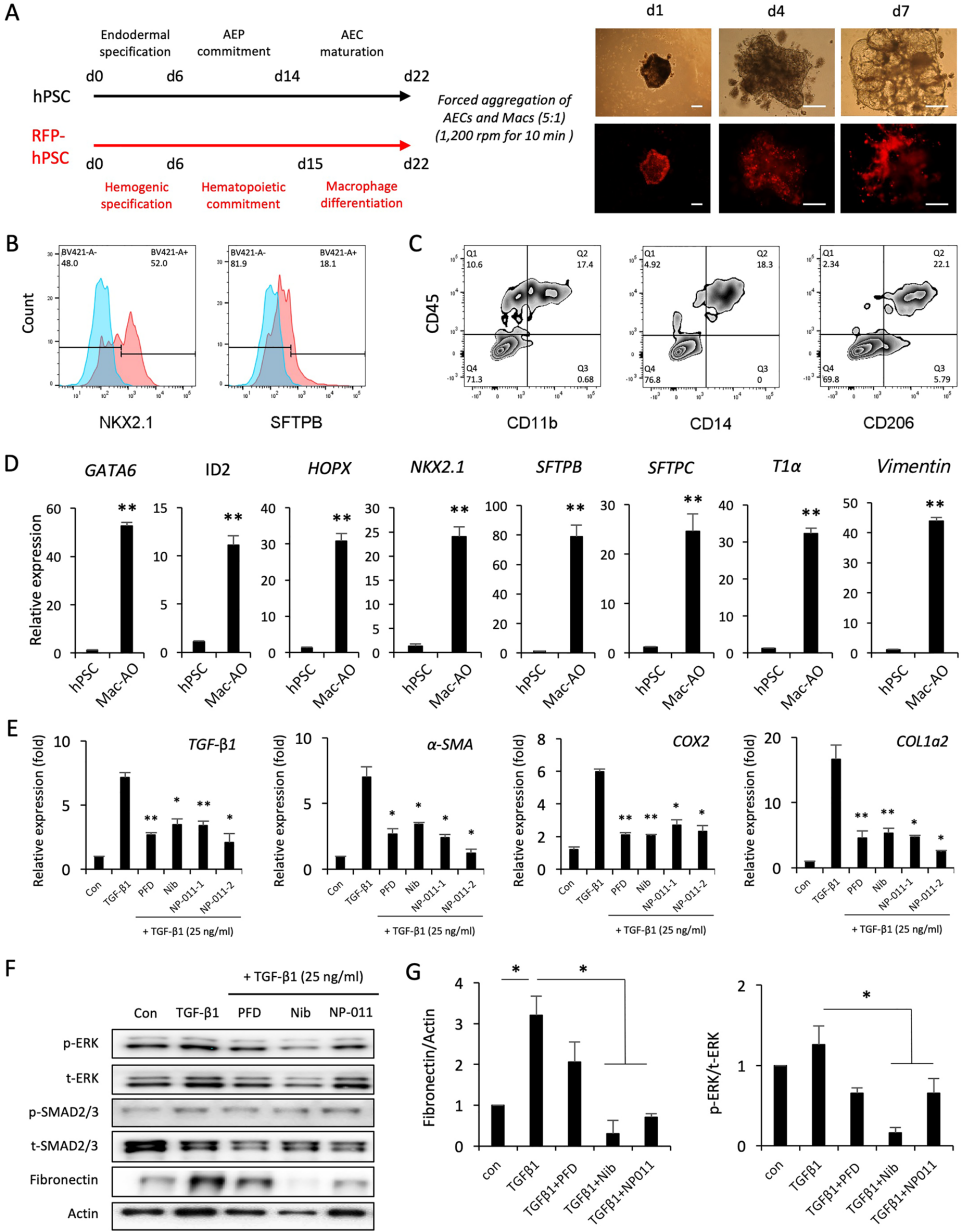
Comparison of anti-fibrotic effects of PFD, Nib, and NP-011 in TGF-β1-induced fibrotic Mac-AOs. **A** Schematic diagram of the generation of Mac-AOs from hPSCs with accompanying representative light and fluorescence microscopy images. Scale bars, 100 μm. **B** and **C** Flow cytometry analysis of the expression of AEC (NKX2.1 and SFTPB) and macrophage (CD11b, CD14 and CD206) markers on Mac-AOs. **D** qPCR of the indicated AEP (GATA6, HOPX, ID2, and NKX2.1), AEC1 (T1α), AEC2 (SFTPB and SFTPC), and mesenchymal stromal cell (Vimentin) markers in Mac-AOs. Data are shown as fold-change relative to undifferentiated hPSCs. **E** Effects of PFD (1 μg/mL), Nib (1 μM), and NP-011 (NP-011-1, 500 ng/mL; NP-011-2, 2 μg/mL) on the expression of fibrosis-related genes in TGF-β1-induced fibrotic Mac-AOs. **F** and **G** Western blotting and subsequent quantification of p-ERK, p-SMAD2/3, and Fibronectin in Mac-AOs from the indicated groups. Actin was used as a loading control. Data are presented as mean ± s.d. *p < 0.05, **p < 0.01

Forced aggregation of functional AECs and macrophages combined with our established differentiation system enabled us to generate Mac-AOs [3, 4]. AECs and macrophages were aggregated at a 5:1 ratio and cultured for 7 days (Additional file 1: Fig. S3). The aggregates formed alveolar sac-like structures with multiple alveoli containing macrophages (Fig. 2A). Flow cytometry analysis revealed that Mac-AOs express AEC (NKX2.1 and SFTPB) and macrophage (CD11b, CD14 and CD206) markers and retain the initial mixing ratio of cells upon aggregation (Fig. 2B and C). We also found that the AEP, ACE1, ACE2, and mesenchymal stromal cells-related genes were highly upregulated in Mac-AOs compared to levels in undifferentiated hPSC cultures (Fig. 2D). These observations confirmed that our method enables the generation of multicellular AOs containing AEPs, AEC1, AEC2, and mesenchymal stromal cells from hPSCs (Additional file 2).

To evaluate the potential use of Mac-AOs for modeling PF and drug efficacy testing, Mac-AOs were exposed to TGF-β1 (25 ng/mL) for 72 h. This exposure induced fibrotic changes, which were significantly reduced by treatment of the Mac-AOs with PFD (1 μg/mL), and Nib (1 μM), and NP-011 (NP-011–1, 500 ng/mL; NP-011–2, 2 μg/mL) (Fig. 2E; Additional file 1: Fig. S4). Furthermore, western blot analysis demonstrated a significant reduction of fibronectin with concomitant suppression of non-canonical extracellular signal-regulated kinase (ERK) signaling in Nib- and NP-011-treated Mac-AOs but not in PFD-treated Mac-AOs (Fig. 2F and G). These results suggest that hPSC-derived Mac-AOs more closely recapitulate the complexity and features of in vivo human alveolar tissues and provide a robust in vitro system for modeling PF and testing drug efficacy (Additional file 2).

Macrophages are remarkably plastic cells that can transform from the pro-inflammatory M1 phenotype to the anti-inflammatory M2 phenotype and vice versa [8]. Internal and external stimuli induce dynamic changes in macrophage phenotype, which is closely associated with either exacerbation or prevention of disease progression [9]. In the pathogenesis of IPF, activated M1 macrophages are thought to act as a double-edged sword to either trigger or inhibit fibrotic responses, and suppression of the activation and recruitment of M2 macrophages therefore may offer a therapeutic strategy [8, 10]. Based on these findings, modulation of macrophage phenotype has been implicated in the pathogenesis of PF. Thus, we generated Mac-AOs using non-activated naïve macrophages to avoid undesirable inflammatory or fibrotic responses prior to fibrosis induction to enable fibrosis response and drug testing. Indeed, no significant differences were detected in the basal expression levels of inflammation- and fibrosis-related genes between AOs and Mac-AOs (data not shown). A recent study also demonstrated a dampened inflammatory phenotype of mouse PSC-derived macrophages compared with BM-derived macrophages [11]. Importantly, in comparison with AOs, TGF-β1 treatment induced stronger fibrotic changes in Mac-AOs, and these changes were effectively reduced by suppression of ERK signaling relevant to M2 macrophage polarization. These results suggest that human and mouse PSC-derived macrophages may be slightly skewed toward an anti-inflammatory M2-like phenotype and would favor fibrotic changes in Mac-AOs. Thus, further investigation to define the mechanism of modulating macrophage phenotypes will enable the use of Mac-AOs in the development of novel effective therapeutics for PF and other fibrosis diseases.

In conclusion, we established a protocol to generate hPSC-derived multicellular Mac-AOs, which could serve as a powerful tool for predicting the therapeutic potential of drug candidate for PF as well as for modeling pulmonary diseases. We assert that this study sets the stage for using functional tissue-specific macrophages (i.e., Kupffer cells, microglia, and gastrointestinal macrophages) as key components of other types of organoids, including liver, brain, and intestine. Indeed, bronchioalveolar lung organoid derived from mouse bronchioalveolar stem cells support engraftment and maintenance of injected alveolar macrophages, which interact with the AECs to drive cell maturation and sense signals from the injured AECs upon infection. This study suggests that macrophage-containing AOs can be used to study macrophage-AEC crosstalk in the context of infection and AO maturation [12]. Furthermore, as a fine mesh of capillaries wraps around each alveolus and covers about 70% of its surface area for gas exchange, integration of key missing vessel components will increase the complexity and functions of our Mac-AOs model and facilitate additional study of pulmonary diseases and associated therapeutics.

## Supplementary Information

**Additional file 1:** **Figure S1.** Generation and characterization of hPSC-derived macrophages. (A) Morphologies of macrophages derived from three hiPSC lines (CMC003, CMC009 and CMC011). Scale bar, 100 μm. (B) Diff-Quik staining of macrophages. Scale bar, 100 μm. (C) Phagocytic cells engulfing green fluorescent beads. Scale bar, 100 μm. (D) Flow cytometry analysis for expression of macrophage markers on macrophages. **Figure S2.** Effect of CyPA on hPSC-derived macrophage differentiation. Flow cytometry analysis for expression of macrophage markers on macrophages cultured in the presence and absence of CyPA. Data are presented as mean±s.d. **Figure S3.** Generation of Mac-AOs from hPSCs. (A) Forced aggregation of AECs (50K cells) and macrophages (10K cells) labeled with PKH26 Red Fluorescent Cell Linker (Sigma, MINI26). (B) Representative images of aggregates and Mac-AO after 24 h and 7 days of aggregation, respectively. Scale bars, 100 μm. **Figure S4.** Immunostaining of collagen in Mac-AOs. Representative images show collagen staining of Mac-AO sections from the indicated groups. Scale bars, 50 μm.

**Additional file 2.** Supplementary materials and methods.

## Data Availability

All datasets supporting the conclusions of this article is included within the article (and its additional files).

## Abbreviations

AEP: Alveolar epithelial progenitors
AEC1: Type 1 alveolar epithelial cell
AEC2: Type 2 alveolar epithelial cell
PFD: Pirfenidone
Nib: Nintedanib
