# Supplementary material for: Generation of macrophage containing alveolar organoids derived from human pluripotent stem cells for pulmonary fibrosis modeling and drug efficacy testing
Source: Cell Biosci. 2021 Dec 18;11:216. doi: 10.1186/s13578-021-00721-2 (PMC8684607; doi:10.1186/s13578-021-00721-2)
Supplement: Supplementary file 1 — Additional file 1: Figure S1. Generation and characterization of hPSC-derived macrophages. (A) Morphologies of macrophages derived from three hiPSC lines (CMC003, CMC009 and CMC011). Scale bar, 100 μm. (B) Diff-Quik staining of macrophages. Scale bar, 100 μm. (C) Phagocytic cells engulfing green fluorescent beads. Scale bar, 100 μm. (D) Flow cytometry analysis for expression of macrophage markers on macrophages. Figure S2. Effect of CyPA on hPSC-derived macrophage differentiation. Flow cytometry analysis for expression of macrophage markers on macrophages cultured in the presence and absence of CyPA. Data are presented as mean±s.d. Figure S3. Generation of Mac-AOs from hPSCs. (A) Forced aggregation of AECs (50K cells) and macrophages (10K cells) labeled with PKH26 Red Fluorescent Cell Linker (Sigma, MINI26). (B) Representative images of aggregates and Mac-AO after 24 h and 7 days of aggregation, respectively. Scale bars, 100 μm. Figure S4. Immunostaining of collagen in Mac-AOs. Representative images show collagen staining of Mac-AO sections from the indicated groups. Scale bars, 50 μm. [file 13578_2021_721_MOESM1_ESM.docx]

**Additional File 1 for**

**Generation of macrophage containing alveolar organoids derived from human pluripotent stem cells for pulmonary fibrosis modeling and drug efficacy testing**

Hye-Ryeon Heo^1^, Seok-Ho Hong^1,2,3^*

^1^ *Department of Internal Medicine, School of Medicine, Kangwon National University, Chuncheon, Republic of Korea*

^2^ *Institute of Medical Science, School of Medicine, Kangwon National University, Chuncheon, Republic of Korea*

^3^ *Environmental Health Center*, *Kangwon National University Hospital, Chuncheon, Republic of Korea*

**
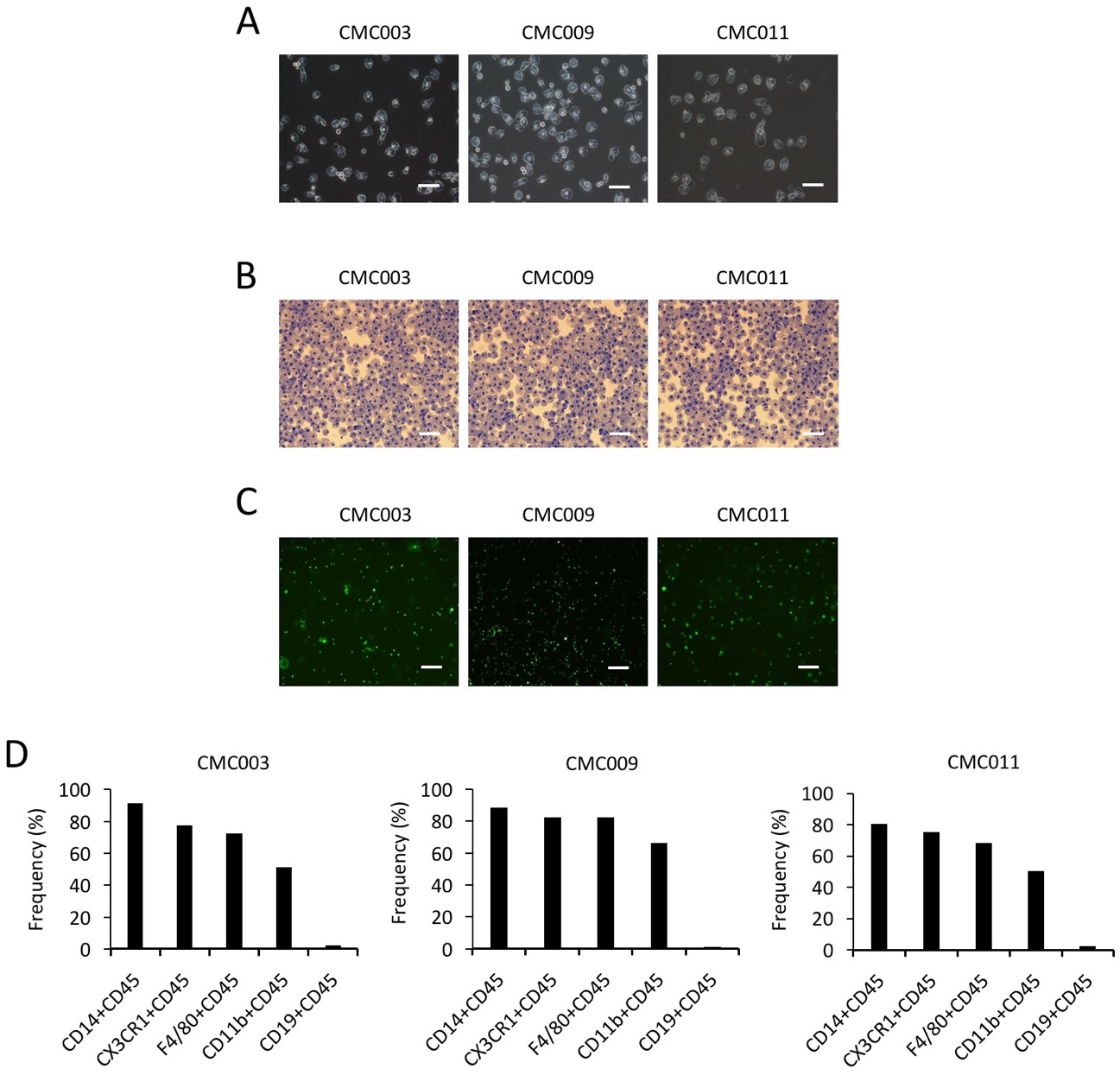
**

**Figure S1. Generation and characterization of hPSC-derived macrophages.** (A) Morphologies of macrophages derived from three hiPSC lines (CMC003, CMC009 and CMC011). Scale bar, 100 μm. (B) Diff-Quik staining of macrophages. Scale bar, 100 μm. (C) Phagocytic cells engulfing green fluorescent beads. Scale bar, 100 μm. (D) Flow cytometry analysis for expression of macrophage markers on macrophages.

**
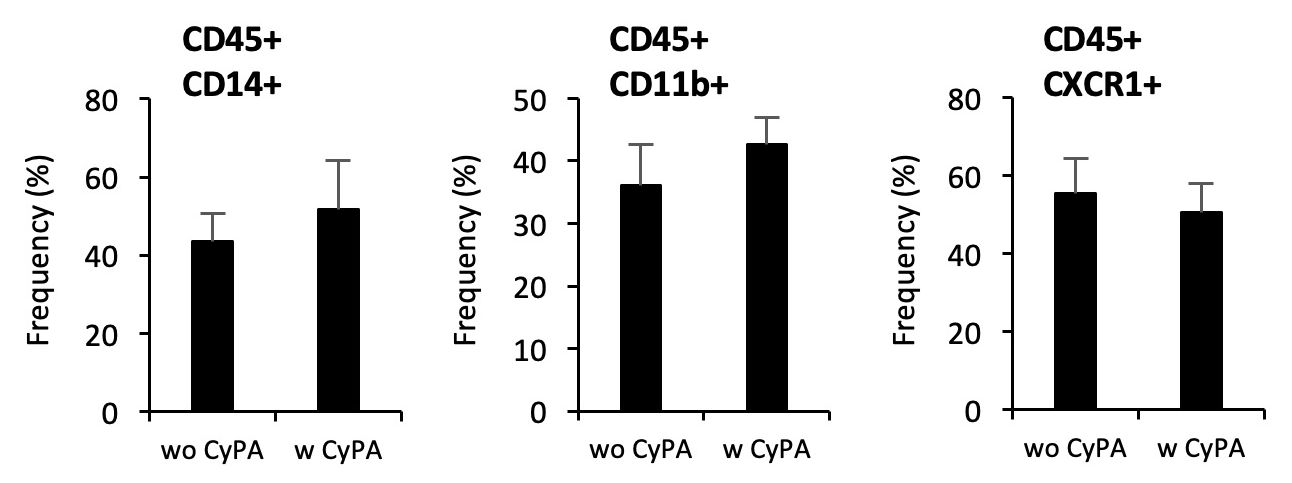
**

**Figure S2. Effect of CyPA on hPSC-derived macrophage differentiation.** Flow cytometry analysis for expression of macrophage markers on macrophages cultured in the presence and absence of CyPA. Data are presented as mean±s.d.

**
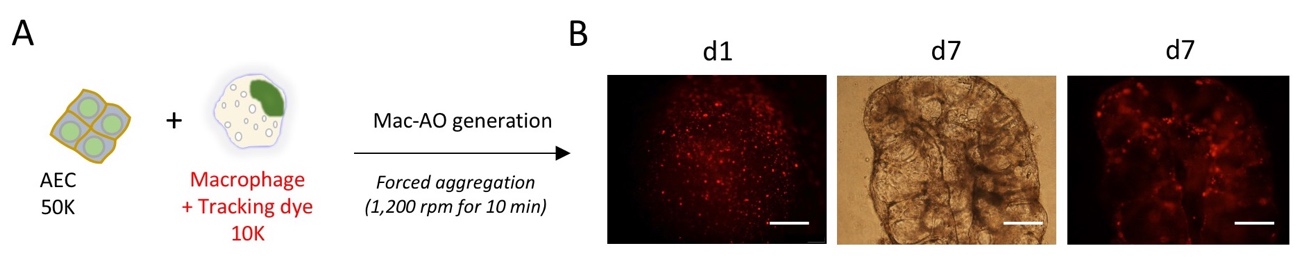
**

**Figure S3. Generation of Mac-AOs from hPSCs.** (A) Forced aggregation of AECs (50K cells) and macrophages (10K cells) labeled with PKH26 Red Fluorescent Cell Linker (Sigma, MINI26). (B) Representative images of aggregates and Mac-AO after 24 h and 7 days of aggregation, respectively. Scale bars, 100 μm.

**
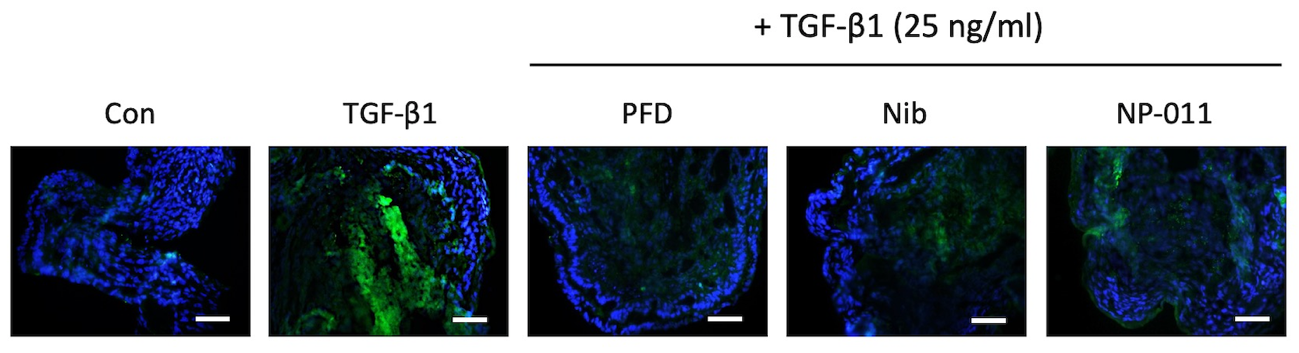
**

**Figure S4. Immunostaining of collagen in Mac-AOs.** Representative images show collagen staining of Mac-AO sections from the indicated groups. Scale bars, 50 μm.
