# Supplementary material for: Generation of macrophage containing alveolar organoids derived from human pluripotent stem cells for pulmonary fibrosis modeling and drug efficacy testing
Source: Cell Biosci. 2021 Dec 18;11:216. doi: 10.1186/s13578-021-00721-2 (PMC8684607; doi:10.1186/s13578-021-00721-2)
Supplement: Supplementary file 2 — Additional file 2. Supplementary materials and methods. [file 13578_2021_721_MOESM2_ESM.docx]

**Additional File 2 for**

**Generation of macrophage containing alveolar organoids derived from human pluripotent stem cells for pulmonary fibrosis modeling and drug efficacy testing**

Hye-Ryeon Heo^1^, Seok-Ho Hong^1,2,3^*

^1^ *Department of Internal Medicine, School of Medicine, Kangwon National University, Chuncheon, Republic of Korea*

^2^ *Institute of Medical Science, School of Medicine, Kangwon National University, Chuncheon, Republic of Korea*

^3^ *Environmental Health Center*, *Kangwon National University Hospital, Chuncheon, Republic of Korea*

**Materials and Methods**

**Maintenance of hPSCs**

Human PSCs (iPS-NT4-S1) were kindly provided by CHA University, South Korea. Additionally, human induced PSCs (CMC003, CMC009, and CMC011) were obtained from Korea National Institute of Health. The cells were cultured under serum-, xeno-, and feeder-free conditions using E8 medium (STEMCELL Technologies) on dishes coated with vitronectin (STEMCELL Technologies). All cell lines were incubated at 37°C in a humidified atmosphere with 5% CO_2._

**AEC differentiation of hPSCs**

A stepwise direct AEC differentiation was performed as previously described (1). Briefly, undifferentiated hPSC colonies were prepared with low density of less than 5 colonies per well. When the colonies grew to approximately 1 mm in diameter, AEC differentiation was initiated with exposure to sequential induction medium.

**Human cells**

Human umbilical cord (hUC) tissues were obtained from full-term births after Caesarian sections with informed consent according to the guidelines approved by the IRB at Kangwon National University Hospital (IRB approval number: KNUH-2012-11-003-008). hUC-PVCs were isolated and cultured, as previously described (2). Human skin fibroblasts (CRL-2522^TM^) and BM-MSCs (PCS-500-012^TM^) were purchased from ATCC and cultured according to the manufacturer’s instructions.

**Macrophage differentiation from hPSCs and CyPA treatment**

Macrophages differentiation was conducted as previously described (3). Briefly, the floating hematopoietic progenitor cells between day 14 to 16 of hematopoietic differentiation and cultured them for 7 days in macrophage induction medium containing RPMI1640 (Gibco, #22400089) supplemented with 10% of fetal bovine serum (FBS, Hyclone), 1% of penicillin/streptomycin (Gibco, 17504-063) and 100 ng/ml macrophage colony-stimulating factor (M-CSF, Peprotech, #300-25). In order to evaluate the effects of cylophilin A (CyPA), hematopoietic progenitor cells were seeded at a density of 6×10^5^ cells/well of 12 well plate and cultured in the presence and absence of CyPA (50 ng/mL) for 8 days. The medium was changed every 2 days.

**Generation of Mac-AOs**

For the generation of Mac-AOs, two-dimensional cultures were dissociated on day 22 of AEC differentiation with 0.4 U/mL collagenase B (Roche) for 2 h in a 37°C incubator, followed by treatment with cell dissociation buffer (Gibco) for 10 min in a 37°C water bath to singularize cells. The single-cell suspension was then passed through a 70-μm cell strainer (BD Bioscience). hPSC-derived macrophages were also harvested on day 22 of differentiation. AECs (5×10^4^ cells) and macrophages (1×10^4^ cells) were seeded into 96-well round-bottom plates (Corning, 6×10^4^ cells per well) containing AEC maturation medium supplemented with 10 μM ROCK inhibitor (STEMCELL Technologies). Macrophages were labeled with CellTracker^TM^ Deep RFP dye prior to seeding for the confirmation of their existence in Mac-AOs. Plates were centrifuged at 1,200 rpm for 10 min and incubated overnight at 37°C in a humidified atmosphere containing 5% CO_2_. After overnight culture, the aggregates were transferred to 6-well low-attachment plates (Corning) containing fresh AEC maturation medium and cultured for 8 days to establish Mac-AOs. Mac-AOs were collected and used in all assays.

**BALF and collection of primary macrophages**

This prospective study included 10 patients [a control group (n=5), and a pulmonary infection group (n = 5)] who underwent a bronchoalveolar lavage (BAL) at the Chuncheon Sacred Heart Hospital between June 2018 and August 2018. All human study protocols were approved by the Chuncheon Sacred Heart Hospital Institutional Review Board (institutional review board number 2017-49). Subjects with non-malignant single granuloma were used as the control group. In the control group, the BAL fluid (BALF) for the study was collected from the bronchus opposite the granuloma. Briefly, 50 ml of 0.9% saline (37°C) was instilled into the bronchus by a flexible bronchofiberscope (Olympus, Japan) and the BALF was recovered. After analyzing the BALF by WBC differential count (XN-3000, Sysmex, Japan), we found that more than 70% of the BALF are human alveolar macrophages (AMs) (4). AMs and hPSC-Macs were seeded at 1x 10^6^ cells per well in 12-well plates and were incubated with serum-free RPMI1640 for 2 h and then were washed to remove detached cells. For the inflammatory response experiment, AMs and hPSC-Macs were cultured in the absence or presence of 100 ng/ml lipopolysaccharide (LPS; Sigma, MO, USA) for 4 h and harvested for further analysis.

**Phagocytosis assay**

Phagocytosis assay was performed as previously described (5). Briefly, two fluorescent beads with different colors, such as green and red, were used in this study (Sigma; #L4655, #L3030). The beads were pre-incubated in RPMI1640 medium supplemented with 10% of human serum for 30 min at 37°C. The beads were incubated with hPSC-Macs (5 μg per 10^4^ cells in 1 ml of macrophage medium) for 2 h at 37°C. The cells were rinsed with cold PBS to remove the non-phagocytosed beads, fixed with 4% paraformaldehyde (PFA, Sigma) and then stained with 4-6-Diamidino-2-phenylindole, dihydrochloride (DAPI, Sigma). The phagocytosis was assessed by a fluorescence microscopy (IX-51, Olympus).

**Diff-Quik staining**

Morphological evaluation of hPSC-Macs was performed according to the manufacturer’s instructions.

**Flow cytometric analysis**

On day 21-23 of differentiation, hPSC-Macs were collected and rinsed with 1% FBS-PBS. Mac-AOs were incubated with collagenase B (Roche, #11088815001) for 2 h, followed by treatment of cell dissociation buffer (Gibco). The cells were passed through a 70 μm cell strainer and resuspended in 1% FBS-PBS. The cells were surface-stained with following fluorochrome-conjugated antibodies for 1 h at 4°C: CD45-APC (1:200 dilution; BD Biosciences, 555485), CD11b (1:10 dilution; Abcam, ab8878), CD11c (1:200 dilution; Invitrogen, MA11C5), CD192-FITC (1:100 dilution; Biolegend, 357216), CX3CR1-FITC (1:100 dilution; Biolegend, 341606), CD14-BV421 (1:100 dilution; BD Biosciences, 563743), CD169 (1:200 dilution; Novus Biologicals, NBP2-30903) and CD206 (1:200 dilution; Novus Biologicals, DDX0380P). For intracellular staining of NKX2.1 and SFTPB, the cells were fixed and permeabilized using the Cytofix/Cytoperm buffer (BD Biosciences). The following primary and secondary antibodies were used: NKX2.1 (1:200 dilution; Abcam, ab76013), SFTPB (1:200 dilution; EMD Millipore), goat anti-mouse Alexa Fluor 488 (Invitrogen, A11001) and goat anti-rabbit Alexa Fluor 488 (Invitrogen, A11034). Dead cells were excluded based on staining with 7-aminoactinomycin D (7-AAD, BD Pharmingen). Flow cytometric analysis was performed by using a FACSCanto^TM^II flow cytometer (BD Bioscience), and acquired data were analyzed with FlowJo software (Tree star).

**RNA extraction and quantitative real-time PCR (qPCR)**

Total RNA was extracted from mouse lung tissues, AOs, Mac-AOs, hPSC-Macs and undifferentiated hPSC cultures using an RNeasy Mini kit (Qiagen, Duesseldorf, Germany) and cDNA was synthesized using TOPscrip^TM^ RT DryMIX (Enzynomics, Daejeon, Korea). PCR amplification was performed using a Step One Plus real time PCR system (Applied Biosystems, Warrington, UK) with TOPreal^TM^ qPCR 2X PreMIX (Enzynomics). All the mRNA expression was normalized to an internal control GAPDH. The primer sequences are listed in Table S1.

**Western blot analysis**

Protein extracted from mouse lung tissues, AOs and human pAECs were lysed in protein lysis buffer and quantified using the BCA protein assay. The 20 μg of protein were separated by SDS-PAGE using 10-12% gel and then transferred to PVDF membranes. Nonspecific binding proteins were blocked with 5% skim milk for 1h at RT. The membranes were incubated with primary antibodies against anti-phospho-p44/42 MAPK, anti-p44/42 MAPK, anti-phospho-Smad2/3, anti-Smad2/3, and anti-fibronectin overnight at 4°C. Membranes were scanned with ChemiDoc imaging system (Bio-Rad Laboratories, Hercules, CA, USA). The antibodies are listed in Table S2.

**Statistical analysis**

Values for all measurements are presented as mean±s.d. Statistical significance was determined using Student’s *t*-test, and *p*<0.05 was considered statistically significant. Three independent experiments were carried out unless otherwise stated.

**References**

1. Heo HR, Kim J, Kim WJ, Yang SR, Han SS, Lee SJ *et al*. Human pluripotent stem cell-derived alveolar epithelial cells are alternatives for in vitro pulmotoxicity assessment. *Sci Rep* 2019;9(1):505.

2. Kim JY, Kim WJ, Ha KS, Han ET, Park WS, Yang SR, Hong SH. Perivascular stem cells supress inflammasome activation during inflammatory responses in macrophages. Int J Stem Cells. 2019;12(3):419-429.

3. Han HY, Seo HH, Jo HY, Han HJ, Falcao V, Delmore V *et al*. Drug discovery platform targeting M. tuberculosis with human embryonic stem cell-derived macrophages. *Stem Cell Rep* 2019

4. [Meyer KC](https://www.ncbi.nlm.nih.gov/pubmed/?term=Meyer%20KC%5BAuthor%5D&cauthor=true&cauthor_uid=22550210), [Raghu G](https://www.ncbi.nlm.nih.gov/pubmed/?term=Raghu%20G%5BAuthor%5D&cauthor=true&cauthor_uid=22550210), [Baughman RP](https://www.ncbi.nlm.nih.gov/pubmed/?term=Baughman%20RP%5BAuthor%5D&cauthor=true&cauthor_uid=22550210), [Brown KK](https://www.ncbi.nlm.nih.gov/pubmed/?term=Brown%20KK%5BAuthor%5D&cauthor=true&cauthor_uid=22550210), [Costabel U](https://www.ncbi.nlm.nih.gov/pubmed/?term=Costabel%20U%5BAuthor%5D&cauthor=true&cauthor_uid=22550210), [du Bois RM](https://www.ncbi.nlm.nih.gov/pubmed/?term=du%20Bois%20RM%5BAuthor%5D&cauthor=true&cauthor_uid=22550210) *et al*. An official American Thoracic Society clinical practice guideline: the clinical utility of bronchoalveolar lavage cellular analysis in interstitial lung disease**.** *Am J Respir Crit Care Med* 2012;185(9):1004-1014.

5. [Sharma L](https://www.ncbi.nlm.nih.gov/pubmed/?term=Sharma%20L%5BAuthor%5D&cauthor=true&cauthor_uid=24908301), [Wu W](https://www.ncbi.nlm.nih.gov/pubmed/?term=Wu%20W%5BAuthor%5D&cauthor=true&cauthor_uid=24908301), [Dholakiya SL](https://www.ncbi.nlm.nih.gov/pubmed/?term=Dholakiya%20SL%5BAuthor%5D&cauthor=true&cauthor_uid=24908301), [Gorasiya S](https://www.ncbi.nlm.nih.gov/pubmed/?term=Gorasiya%20S%5BAuthor%5D&cauthor=true&cauthor_uid=24908301), [Wu J](https://www.ncbi.nlm.nih.gov/pubmed/?term=Wu%20J%5BAuthor%5D&cauthor=true&cauthor_uid=24908301), [Sitapara R](https://www.ncbi.nlm.nih.gov/pubmed/?term=Sitapara%20R%5BAuthor%5D&cauthor=true&cauthor_uid=24908301) *et al*. Assessment of phagocytic activity of cultured macrophages using fluorescence microscopy and flow cytometry. *Methods Mol Biol* 2014;1172:137-145.

**Table S1.** Human Primer sequences used for qPCR

| Genes |  | Sequence 5' to 3' | Product size (bp) |
| --- | --- | --- | --- |
| *T1α* | F | TGC GAA AAA TGT CGG GAA GG | 51 |
|  | R | GGC GTA ACC CTT CAG CTC TT |  |
| *SFTPB* | F | GCC ATA CCA CAG GCA ATG CT | 80 |
|  | R | TGC TGC TCC ACA AAT TGC TT |  |
| *SFTPC* | F | CCT TCT TAT CGT GGT GGT GGT | 96 |
|  | R | TCT CCG TGT GTT TCT GGC TCA T |  |
| *GATA6* | F | CAG CAA AAA TAC TTC CCC CA | 107 |
|  | R | ACT TGA GCT CGC TGT TCT CG |  |
| *HOPX* | F | GCC TTT CCG AGG AGG AGA C | 97 |
|  | R | TCT GTG ACG GAT CTG CAC TC |  |
| *NKX2.1* | F | AGC ACA CGA CTC CGT TCT CA | 75 |
|  | R | CCT CCA TGC CCA CTT TCT TG |  |
| *α-SMA* | F | GAC GAA GCA CAG AGC AAA AG | 70 |
|  | R | AGT TGG TGA TGA TGC CAT GT |  |
| *COL1a1* | F | AAG GGT GAG ACA GGC GAA CA | 70 |
|  | R | GAC CCT GGA GGC CAG AGA AG |  |
| *TGF-β1* | F | AGC AAC AAT TCC TGG CGA TA | 90 |
|  | R | CAC AAC TCC GGT GAC ATC AA |  |
| *IL-6* | F | AGC CCT GAG AAA GGA GAC AT | 175 |
|  | R | TGG AAG GTT CAG GTT GTT TT |  |
| *IL-8* | F | GTG CAG TTT TGC CAA GGA GT | 187 |
|  | R | CTC TGC ACC CAG TTT TCC TT |  |
| *IL-1β* | F | CTG TCC TGC GTG TTG AAA GA | 179 |
|  | R | TTC TGC TTG AGA GGT GCT GA |  |
| *IL-1α* | F | ATC AGT ACC TCA CGG CTG CT | 115 |
|  | R | TGG GTA TCT CAG GCA TCT CC |  |
| *GAPDH* | F | GGC ATG GAC TGT GGT CAT GA | 87 |
|  | R | TGC ACC ACC AAC TGC TTA GC |  |

**Table S2.** Antibodies used for Western blot

| Antibodies | Type | Catalog number | Manufacturer |
| --- | --- | --- | --- |
| p-ERK | rabbit | #4370 | Cell Signaling |
| t-ERK | rabbit | #4695 | Cell Signaling |
| p-SMAD2/3 | rabbit | #8828 | Cell Signaling |
| t-SMAD2/3 | mouse | sc-133098 | Santa Cruz |
| β-Actin | mouse | sc-47778 | Santa Cruz |
| Fibronectin | mouse | sc-59826 | Santa Cruz |
| CyPA | Mouse | Ab58144 | Abcam |
| GAPDH | rabbit | #2118 | Cell Signaling |
